# A Comparison of the Survival, Place of Death, and Medical Utilization of Terminal Patients Receiving Hospital-Based and Community-Based Palliative Home Care: A Retrospective and Propensity Score Matching Cohort Study

**DOI:** 10.3390/ijerph18147272

**Published:** 2021-07-07

**Authors:** Cheng-Pei Lin, Min-Shiow Tsay, Yi-Hui Chang, Hung-Cheng Chen, Ching-Yu Wang, Yun-Shiuan Chuang, Chien-Yi Wu

**Affiliations:** 1 Institute of Community Health Care, College of Nursing, National Yang Ming Chiao Tung University, Taipei 112, Taiwan; cheng-pei.lin@nycu.edu.tw; 2 Cicely Saunders Institute, Florence Nightingale Faculty of Nursing, Midwifery and Palliative Care, King’s College London, London SE5 9PJ, UK; 3 Department of Nursing, Kaohsiung Medical University Hospital, Kaohsiung Medical University, Kaohsiung 807, Taiwan; minshow0644@gmail.com (M.-S.T.); 870140@kmuh.org.tw (Y.-H.C.); olive575589@gmail.com (H.-C.C.); 4 Social Work Office, Kaohsiung Medical University Hospital, Kaohsiung Medical University, Kaohsiung 807, Taiwan; 1030150@kmuh.org.tw; 5 Department of Family Medicine, Kaohsiung Medical University Hospital, Kaohsiung Medical University, Kaohsiung 807, Taiwan; kinkipag@gmail.com; 6 Department of Public Health, College of Health Sciences, Kaohsiung Medical University, Kaohsiung 807, Taiwan

**Keywords:** community-based palliative home care, hospital-based palliative home care, survival, place of death, medical utilization, Taiwan

## Abstract

Evidence shows that community-based palliative home care (PHC) provision enhances continuous care and improves patient outcomes. This study compared patient survival, place of death, and medical utilization in community- versus hospital-based PHC. A retrospective cohort study was conducted of patients aged over 18 referred to either community- or hospital-based PHC from May to December 2018 at a tertiary hospital and surrounding communities in Southern Taiwan. A descriptive analysis, Chi-square test, *t*-test, and Log-rank test were used for the data analysis of 131 hospital-based PHC patients and 43 community-based PHC patients, with 42 paired patient datasets analyzed after propensity score matching. More nurse visits (*p* = 0.02), fewer emergency-room visits (*p* = 0.01), and a shorter waiting time to access PHC (*p* = 0.02) were found in the community group. There was no difference in the duration of survival and hospitalization between groups. Most hospital-based patients (57%) died in hospice wards, while most community-based patients died at home (52%). Community-based PHC is comparable to hospital-based PHC in Taiwan. Although it has fewer staffing and training requirements, it is an alternative for terminal patients to meet the growing end-of-life care demand.

## 1. Introduction

The aging population is rapidly increasing globally. According to the United Nations World Population Aging report 2019, there were approximately 703 million people aged over 65 years worldwide in 2019, and this figure is projected to double to 1.5 billion in 2050 [1]. In particular, eastern and south-eastern Asia, Latin America, and the Caribbean are deemed to have the fastest population aging rate, with the percentage of the aged population doubling from 1990 to 2019 (i.e., eastern and south-eastern Asia: 6% to 11%; Latin America and the Caribbean: 5% to 9%) [1].

In Taiwan, the aging rate has surpassed 14%, becoming an “aged society” in 2018, and is projected to become a “super-aged society” in 2025, with 4.7 million Taiwanese people aged 65 years old or over [2]. A global projection study by Sleeman et al. [3,4] reported that the burden of severe health-related suffering among cancer patients is projected to increase rapidly for the aging population (>70 years old). By 2060, 67% of global deaths (approximately 16.3 million) will be accompanied by severe health-related suffering, hence the increasing need for palliative care for symptom control, psychological support, and bereavement care [3]. 

Population aging will put increased financial pressure on old-age support and the healthcare system [1,3]; hence, it is a major global public health challenge as well as a political issue [1,5]. Palliative care has been identified as a core component to respond to older adults’ complex needs [6], as older adults tend to have comorbidities toward the end of their lives, thereby requiring a wide range of physical, psychological, and spiritual healthcare needs [1,5,6]. Integrating palliative care into primary care is crucial to meet the aforementioned needs and improve outcomes (e.g., reduce emergency attendance, increase home death, and improve life quality) for those with a life-threatening illnesses or health-related suffering [7,8]. Evidence shows that community-based palliative home care (PHC) can be beneficial for older adults by improving cost-effective healthcare with better medical utilization and stakeholders’ satisfaction on care, reducing hospital admission and transition from home to hospitals, and supporting home death with a better quality end of life [9,10,11]. Successful integration of community-based PHC with routine care can enhance continuous care through the patient’s illness trajectory from hospital-based care back home. Importantly, community-based services could share the burden of the overloaded inpatient services’ provision for the increasing aging population [12,13].

In Taiwan, palliative care is provided in hospitals or via hospital-based PHC (namely, type A PHC). Hospital-based PHC staff are experienced palliative care professionals including physicians, home care nurses, social workers, and psychologists. To expand PHC services to accommodate the increasing care needs of the aging population, the Ministry of Health and Welfare in Taiwan devised community-based PHC services (namely, type B PHC) in 2014 [14] to enhance the accessibility to palliative care in the community by easing the staff training requirements to improve overall health coverage. The community PHC teams comprise community physicians and home care nurses with back-up from experienced hospital palliative care teams for complex cases. Type A and type B services shared similar patient enrolment criteria and service components, but have different staff training requirements (i.e., type A: 40 h of lectures and 40 h clinical internship with 20 h continuing education annually; type B: 13 h of lectures and 8 h clinical internship with 4 h continuing education annually) and reimbursement. However, the care quality of type B services is questioned due to the perception of insufficient training and practice for patient management, so more PHC patients are cared for by hospital-based PHC teams [14]. Therefore, it was hypothesized that hospital-based PHC would have better patient survival, less acute end-of-life medical utilization, such as emergency-room visits and hospitalization, and a higher proportion of home deaths than community-based PHC.

This is relevant to Taiwan, as the aging population is increasing rapidly; however, there is little evidence regarding community-based PHC in the Taiwanese context. The Ministry of Health and Welfare in Taiwan funded hospitals to develop a community-based PHC model to meet the increasing PHC needs in 2018 [15] and this study is part of the aforementioned program, aiming to compare community-based PHC to hospital-based PHC in terms of patient survival, place of death, and medical utilization in southern Taiwan.

## 2. Materials and Methods

### 2.1. Study Design

This is a retrospective cohort study analyzing data from patients receiving hospital-based PHC and community-based PHC at a tertiary hospital in southern Taiwan from 1 May to 31 December 2018. From our clinical experience, most patients die within a year after receiving PHC; therefore, we followed up the study participants for one year and set the study endpoint as 31 December 2019. The clinical data of deceased patients from both groups were collected until the endpoint for comparison of the duration of their survival, place of death, and medical utilization at a tertiary hospital in southern Taiwan. Strengthening the Reporting of Observational Studies in Epidemiology (STROBE) guidance was adopted for reporting [16]. 

### 2.2. Study Population and Settings

Hospital-based PHC is provided by a multi-disciplinary team in a tertiary university hospital, comprising four physicians, twenty hospice and palliative care unit nurses, three hospice-combined care nurses, three hospice home care nurses, one chaplain, one social worker, one clinical psychologist, and one case manager. There are twenty inpatient beds in the hospice and palliative care unit that provide symptom control, emotional support, and distress relief, incorporating community and home care. Four physicians and three hospice home care nurses within the team primarily provide hospital-based PHC. Community-based PHC is provided by local community clinics run by four physicians and four community home care nurses. Patients aged ≧18 years old diagnosed with any disease receiving either hospital-based or community-based service were deemed as the target cohort for intended outcome comparison. Patients were excluded if they were younger than 18 years old, if their residential area was out of the 10 km radius of the community-based services, or if they were alive at the study endpoint. 

### 2.3. Group Allocation Process

**Figure 1:**
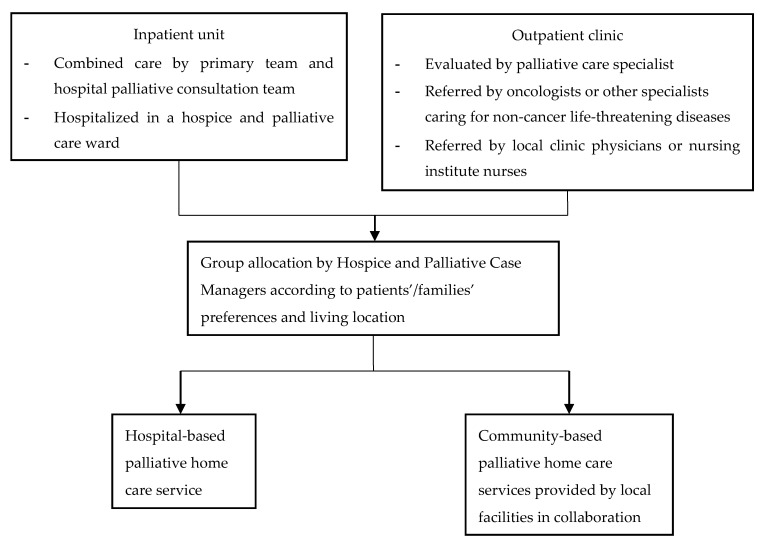
Study group allocation flowchart.

Palliative care inpatient patients were suggested by the hospice and palliative care case managers to receive either hospital-based or community-based services during patient discharge. Patients at outpatient clinics were referred by primary care teams (e.g., hospital oncologists or local community clinic physicians or nurses) to hospital palliative care specialists (e.g., palliative care physicians) for eligibility assessment and were then referred to the hospice and palliative care case managers for suggestions on group allocation. Patients and their family members were provided with information regarding service content, health insurance model, and out-of-pocket payment (e.g., transportation expenses for home visits) by hospital palliative care specialists. The patients and their family members’ preferences and the distance from patient homes to the hospital were considered to inform the recommendations of care received (Figure 1).

### 2.4. Data Sources and Data Collection

The hospital’s electronic medical record system was used to retrieve data for the hospital-based group. Relevant information in the community was provided by the community team in the local clinics or facilities. Patients’ demographic characteristics (e.g., age, gender, educational level, marriage status, religion, terminal disease, and existing wound or tubes), medical utilization (e.g., frequency of physician and nurse visits, visiting time, caring days, frequency of emergency visits, frequency re-admission, hospitalization days), place of death, and duration of survival were recorded. 

### 2.5. Data Analysis

The data were entered into Excel sheets and imported into SPSS 20.0^®^ (SPSS Inc., Chicago, IL, USA) for data management and analysis. Descriptive analysis was used to report patient demographic characteristics by presenting frequency and percentage for categorical data and mean and standard deviation for continuous data. The Chi-square test was used to determine group differences for categorical data and an independent *t*-test was used for continuous data. Furthermore, the Log-rank test was used to assess the difference in survival days between groups. One-to-one propensity score matching (PSM) for control of selection bias and sample size justification was adopted to have the same proportions of the two groups. Variables included in the PSM model were age, gender, religion, Eastern Cooperative Oncology Group (ECOG) score, and terminal diagnosis. The statistical analyses were performed among groups before and after PSM.

### 2.6. Ethical Consideration

Ethical approval was granted by the Kaohsiung Medical University Hospital (KMUH) Institutional Review Board (IRB Number: KMUHIRB-E(I)-20190225) as this is a retrospective anonymous secondary data analysis. Consent forms were not required given the retrospective nature of the study design. 

## 3. Results

### 3.1. Participant Characteristics

**Figure 2:**
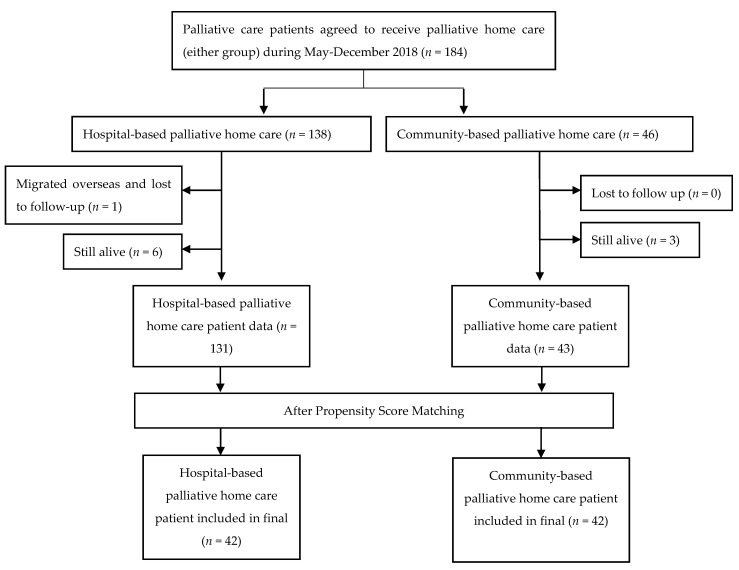
Patient selection flowchart.

A total of 184 patients were eligible for this study, 138 hospital-based patients and 46 community-based patients. After the application of the inclusion criteria, there were 131 hospital-based patients and 43 community-based patients, with 42 paired patient datasets entered for final data analysis after PSM (Figure 2). The demographic results are shown in Table 1, with no significant difference between groups for age, gender, education, marriage, religion, ECOG, terminal diagnosis, and devices/wounds/infusions before and after PSM. The distance from patients’ homes to the hospital significantly influences their assignment for community-based services (*p* < 0.01).

### 3.2. Medical Utilization, Place of Death, and Survival Days

**Figure 3:**
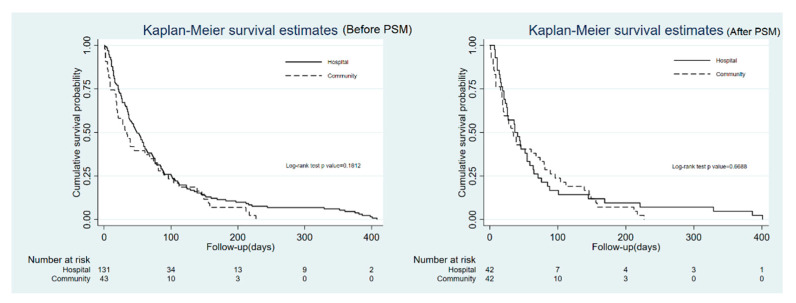
Comparison of the duration of survival between patients receiving hospital-based palliative care and community-based palliative home care before and after PSM.

Before PSM, patients in the hospital-based group received more physician visits (*p* < 0.01) and had longer nurse visit intervals (*p* < 0.01), as well as a longer average length of hospital stay (*p* = 0.03). However, more nurse visits (*p* = 0.01), shorter waiting time to access PHC (*p* < 0.01), and fewer emergency-room visits (*p* = 0.04) were found in the community-based group. There is no statistical difference in physician visit interval, total caring days, number of hospitalizations, and total length of hospital stay (Table 2) as well as survival days (*p* = 0.18) (Figure 3) between the two groups. After PSM, the trend was similar, except for the number of physician visits (*p* = 0.07) and the average length of hospital stay (*p* = 0.08). However, a significant difference in patient place of death was found (*p* = 0.02), with most patients in the hospital-based group tending to die in an inpatient hospice (57%), whereas patients in the community-based group were more likely to die at home (52%) (Table 2).

## 4. Discussion

Unlike previous studies [9,10,17,18,19], this study was the first to compare the terminal patients’ survival, place of death, and medical utilization of patients receiving hospital-based versus community-based PHC in southern Taiwan. The results suggest that community-based PHC can provide timely nurse visits, shorten the waiting time for patients to PHC, reduce emergency visits, and facilitate home death, with no significant difference in patients’ caring days, survival days, and hospitalization rates between groups. Furthermore, people who lived far away from the healthcare facilities (e.g., nursing home care institutes or hospitals) were more likely to receive community-based PHC. 

It is noteworthy in the hospital-based group that the visit frequency of nurses and physicians was similar (nurse versus physicians: mean ± SD: 5.57 ± 6.95 versus 5.17 ± 6.56), whereas the community-based group received more nurse visits (nurses versus physicians: mean ± SD: 10.19 ± 9.99 versus 3.14 ± 2.40). This might be explained by the different staffing levels in the two care models. For example, the hospital team usually pair a physician and a nurse to visit patients, whereas, in the community-based model, physicians or nurses often visit the patients alone, sharing patient information afterwards for continuous care provision. Therefore, community-based services are considered more flexible and accessible, with timely care provision, which might correlate with the findings in terms of shorter waiting times to access PHC services, fewer emergency-room visits, and timely symptom management. Our findings are comparable with studies conducted in Italy [20,21] and Spain [19] for cancer patients and studies in the USA for home hospice patients [22], indicating that community-based PHC would reduce the frequency of emergency-room visits. Subsequently, patients are more likely to die at home. Cai et al. [23]. reported similar findings for cancer patients in Canada that more home-based nursing visits in the community could increase the congruence between the preferred and actual places of death, with the home being the most preferred place of death. This phenomenon exists not only in the West but also in Asian countries such as Japan [24] and Taiwan [25].

No significant difference in patient survival days was found between the two PHC models during the study period, indicating that the community-based PHC team provides comparable end-of-life care to hospital-based services, even though staff received fewer training hours. In Taiwan, community-based PHC teams may consult hospital-based palliative care teams for complex cases, which might explain the aforementioned findings. The collaboration between the hospital and community teams could increase access to palliative care for all, improve the quality of care transition, and enhance the continuous care provision during a patient’s disease trajectory from diagnosis to death [26,27]. 

Taken together, these findings demonstrate that community-based PHC has the potential to provide comparable quality end-of-life care for patients and their family members compared to hospital-based PHC. Since 2015, the Taipei City medical system of Taiwan has advocated and promoted community- and home-based palliative care. They constructed a friendly and supportive healthcare system for health-promoting palliative care and encouraged community medical teams to involve end-of-life care. This strategy echoes our findings and is hoped to facilitate community end-of-life care provision and improve death literacy [28]. Additionally, community-based PHC could offer services to more people in a wider region, thereby improving the accessibility for people living in rural areas with difficulties accessing hospital care, saving costs, and facilitating appropriate medical resource reallocation. We suggest establishing a collaborative network of various services (i.e., inpatient, outpatient, and community services) to improve universal health coverage and respond to the growing care demand of the increasing aging population in this era of medical resource scarcity. The success of such collaboration has been identified worldwide. For example, in the UK, the collaboration between hospital and community palliative care teams increased palliative care case referrals and improved early access to palliative care services for patients [29]. In the Netherlands, the home care provided jointly by the hospice team, primarily general practitioners and district nurses, increased the possibility of patient death in their preferred locations [30]. Additionally, integrated palliative care services by inpatient, outpatient, and home care have been used to meet patients’ complex end-of-life care needs in the USA [31]. 

This study has several strengths. First, it is one of the first to compare patient outcomes of different PHC models for terminal patients in Taiwan. The findings promote the importance of community PHC provision and inform the policy regarding medical resource reallocation for people with palliative care needs in the community. Second, the low attrition rate is highlighted, which is rare in palliative care research, given the fact that the participants are often very frail and vulnerable. This might be explained by the proactive approach of healthcare staff, as well as trustful clinician–patient relationships. Third, we performed PSM to minimize the selection bias. However, there are limitations to adopting the findings in practice. First, the small sample size after PSM might limit the generalizability of the findings. Second, the patients’ emergency-room visits and medical utilization might be underestimated as we were unable to determine if they sought healthcare services from other healthcare facilities during the study period. Third, we cannot conduct a cost-effectiveness analysis due to the limited data collated. For example, we only collected the frequency of clinician visits, not the cost and total length of time spent on palliative home care provision.

## 5. Conclusions

This study demonstrates that community-based PHC can provide palliative care comparable to hospital-based services to improve outcomes, even though community-based PHC requires less staffing and fewer training hours. Therefore, for terminal patients with end-of-life care needs, community-based PHC is an alternative to hospital-based PHC and should be promoted to meet the growing care demands. However, more research on the feasibility and effectiveness of implementing community-based services and cost evaluation is needed before widely integrating such services into routine practice. 

## Figures and Tables

**Table 1:** Demographic and clinical characteristics of enrolled patients.

Variables	Palliative Home Care Type (Before PSM)		*p*-Value	Palliative Home Care Type (After PSM)		*p*-Value
	Hospital (*n* = 131)	Community (*n* = 43)		Hospital (*n* = 42)	Community (*n* = 42)	
	N (%)/Mean ± SD			N (%)/Mean ± SD		
Age	74.38 ± 12.30	72.14 ± 12.81	0.31	71.33 ± 13.46	72.31 ± 12.92	0.74
Gender			0.60			0.66
Female	79 (60.3%)	24 (55.8%)		22 (52.4%)	24 (57.1%)	
Male	52 (39.7%)	19 (44.2%)		20 (47.6%)	18 (42.9%)	
Education			0.99			0.52
≦6 years	70 (53.4%)	23 (53.5%)		18 (42.9%)	23 (54.8%)	
7~12 years	49 (37.4%)	16 (37.2%)		20 (47.6%)	15 (35.7%)	
>12 years	12 (9.2%)	4 (9.3%)		4 (9.5%)	4 (9.5%)	
Marriage			0.98			0.47
Single	8 (6.1%)	2 (4.7%)		3 (7.1%)	2 (4.8%)	
Married	68 (51.9%)	22 (51.2%)		27 (64.3%)	21 (50.0%)	
Widowed	47 (35.9%)	16 (37.20%)		10 (23.8%)	16 (38.1%)	
Separated/ Divorced	8 (6.1%)	3 (7.0%)		2 (4.8%)	3 (7.1%)	
Religion			0.37			0.72
None	33 (25.2%)	10 (23.3%)		12 (28.6%)	10 (23.8%)	
Buddhism	43 (32.8%)	12 (27.9%)		12 (28.6%)	12 (28.6%)	
Christianity	10 (7.6%)	2 (4.7%)		4 (9.5%)	2 (4.8%)	
Taoism/Folk religion	45 (34.4%)	18 (41.9%)		14 (33.3%)	18 (42.9%)	
Other	0 (0%)	1 (2.3%)		0 (0%)	0 (0%)	
ECOG			0.23			0.25
2	1 (0.8%)	2 (4.7%)		0 (0%)	2 (4.8%)	
3	66 (50.4%)	20 (46.5%)		24 (57.1%)	19 (45.2%)	
4	64 (48.9%)	21 (48.8%)		18 (42.9%)	21 (50%)	
Terminal Diagnosis			0.64			0.99
Cancer	117 (89.3%)	42 (97.7%)		41 (97.6%)	41 (97.6%)	
Brain disease	7 (5.3%)	1 (2.3%)		1 (2.4%)	1 (2.4%)	
Organ failure	6 (4.6%)	0 (0%)		0 (0%)	0 (0%)	
ALS	1 (0.8%)	0 (0%)		0 (0%)	0 (0%)	
Devices/Wounds/Infusion ^1^						
Nasogastric tube	41 (31.3%)	12 (27.9%)	0.68	10 (23.8%)	12 (28.6%)	0.62
Foley tube	51 (38.9%)	15 (34.9%)	0.64	17 (40.5%)	15 (35.7%)	0.65
Tracheostomy	3 (2.3%)	1 (2.3%)	0.99	1 (2.4%)	1 (2.4%)	0.99
Gastro-/Jejunostomy	6 (4.6%)	3 (7.0%)	0.69	2 (4.8%)	3 (7.1%)	0.99
Colostomy	13 (9.9%)	3 (7.0%)	0.76	3 (7.1%)	3 (7.1%)	0.99
Intravenous Fluid	8 (6.1%)	0 (0%)	0.20	3 (7.1%)	0 (0%)	0.24
Wound	42 (32.1%)	14 (32.6%)	0.95	13 (31.0%)	14 (33.3%)	0.82
Distance from nursing institution to patient’s home (km)	3.92 ± 2.12	8.21 ± 5.23	<0.01	3.71 ± 1.92	7.90 ± 4.86	<0.01
Distance from hospital to patient’s home (km)	3.92 ± 2.23	9.29 ± 7.35	<0.01	3.71 ± 1.92	8.72 ± 6.39	<0.01

Data presented as mean ± standard deviation (SD) for continuous variables and frequency (percentage, %) for categorical variables. The *p*-values were calculated using the *t*-test for continuous variables and the Chi-square test for categorical variables. ^1^ Multiple selection is allowed. PSM: Propensity Score Matching; ECOG: Eastern Cooperative Oncology Group; ALS: Amyotrophic Lateral Sclerosis.

**Table 2:** Medical utilization condition of enrolled patients after receiving PHC.

Variables	Palliative Home Care Type (Before PSM)		*p*-Value	Palliative Home Care Type (After PSM)		*p*-Value
	Hospital (*n* = 131)	Community (*n* = 43)		Hospital (*n* = 42)	Community (*n* = 42)	
	N (%)/Mean ± SD			N (%)/Mean ± SD		
Number of physician visits	5.18 ± 5.46	3.09 ± 2.39	<0.01	5.17 ± 6.56	3.14 ± 2.40	0.07
Physician visit interval (day/time)	15.59± 9.40	18.28 ± 15.63	0.29	15.00 ± 9.32	18.6 ± 15.61	0.20
Number of nurse visits	5.91 ± 5.93	9.98 ± 9.97	0.01	5.57 ± 6.95	10.19 ± 9.99	0.02
Nurse visit interval (day/time)	13.07± 8.66	6.30 ± 3.65	<0.01	14.19 ± 9.25	6.40 ± 3.63	<0.01
Total days of care	82.12 ± 95.38	61.30 ± 64.40	0.18	71.76 ± 95.5	62.71 ± 64.5	0.61
The wait time between referral and acceptance to PHC	9.53 ± 5.87	6.47 ± 5.41	<0.01	9.45 ± 6.43	6.40 ± 5.46	0.02
Ever been to ED after referred to PHC but not yet received PHC			0.06			0.04
Yes	21 (16%)	2 (4.7%)		8 (19.0%)	2 (4.8%)	
No	110 (84%)	41 (95.3%)		34 (81.0%)	40 (95.2%)	
The total number of ED visits after referred to PHC but not yet received PHC	0.18 ± 0.420	0.07 ± 0.33	0.10	0.21 ± 0.47	0.07 ± 0.34	0.12
Ever been to the ED			0.049			0.01
Yes	86 (65.6%)	21 (48.8%)		31 (73.8%)	20 (47.6%)	
No	45 (34.4%)	22 (51.2%)		11 (26.2%)	22 (52.4%)	
Number of ED visits	0.90 ± 0.927	0.58 ± 0.698	0.04	1.07 ±0.92	0.57 ±0.70	<0.01
Ever been hospitalized			0.73			0.36
Yes	80 (61.1%)	25 (58.1%)		29 (69.0%)	25 (59.5%)	
No	51 (38.9%)	18 (41.9%)		13 (31.0%)	17 (40.5%)	
Number of hospitalizations	0.78 ± 0.797	0.77 ± 0.782	0.94	0.90 ± 0.85	0.79 ± 0.72	0.51
The total length of hospital stays	9.92 ± 13.59	7.23 ± 10.71	0.24	10.40 ± 12.54	7.40 ± 10.78	0.24
The average length of hospital stay	7.84 ± 10.12	5.03 ± 6.12	0.03	8.42 ± 10.32	5.15 ± 6.14	0.08
Place of death			0.13			0.02
Home	47 (35.9%)	22 (51.2%)		12 (28.6%)	22 (52.4%)	
Nursing/residential home	8 (6.1%)	1 (2.3%)		3 (7.1%)	1 (2.4%)	
Palliative care unit/inpatient hospice	63 (48.1%)	16 (37.2%)		24 (57.1%)	16 (38.1%)	
General wards	3 (2.3%)	3 (7.0%)		0 (0%)	3 (7.1%)	
Emergency department	10 (7.6%)	1 (2.3%)		3 (7.1%)	0 (0%)	

Data presented as mean ± standard deviation (SD) for continuous variables and frequency (percentage, %) for categorical variables. The *p*-values were calculated using the *t*-test for continuous variables and the Chi-square test for categorical variables. PSM: Propensity Score Matching; PHC: palliative home care; ED: emergency department.

## Data Availability

The data presented in this study are available on request from the corresponding author. The data are not publicly available due to privacy.
